# Variability in H9N2 haemagglutinin receptor-binding preference and the pH of fusion

**DOI:** 10.1038/emi.2016.139

**Published:** 2017-03-22

**Authors:** Thomas P Peacock, Donald J Benton, Jean-Remy Sadeyen, Pengxiang Chang, Joshua E Sealy, Juliet E Bryant, Stephen R Martin, Holly Shelton, John W McCauley, Wendy S Barclay, Munir Iqbal

**Affiliations:** 1 Avian Viral Diseases Programme, The Pirbright Institute, Pirbright Woking, GU24 0NF, UK; 2 Imperial College London, London W2 1NY, UK; 3 The Francis Crick Institute, London NW1 1AT, UK; 4 Royal Veterinary College, University of London, London NW1 0TU, UK; 5 Oxford University Clinical Research Unit and Wellcome Trust Major Overseas Programme, National Hospital of Tropical Diseases, 78 Giai Phong, Dong Da, Hanoi, Vietnam; 6 Structural Biology Science Technology Platform, The Francis Crick Institute, London NW1 1AT, UK

**Keywords:** avian influenza, haemagglutinin, H9N2, receptor binding, stability, sulphated, zoonotic

## Abstract

H9N2 avian influenza viruses are primarily a disease of poultry; however, they occasionally infect humans and are considered a potential pandemic threat. Little work has been performed to assess the intrinsic biochemical properties related to zoonotic potential of H9N2 viruses. The objective of this study, therefore, was to investigate H9N2 haemagglutinins (HAs) using two well-known correlates for human adaption: receptor-binding avidity and pH of fusion. Receptor binding was characterized using bio-layer interferometry to measure virus binding to human and avian-like receptor analogues and the pH of fusion was assayed by syncytium formation in virus-infected cells at different pHs. We characterized contemporary H9N2 viruses of the zoonotic G1 lineage, as well as representative viruses of the zoonotic BJ94 lineage. We found that most contemporary H9N2 viruses show a preference for sulphated avian-like receptor analogues. However, the ‘Eastern' G1 H9N2 viruses displayed a consistent preference in binding to a human-like receptor analogue. We demonstrate that the presence of leucine at position 226 of the HA receptor-binding site correlated poorly with the ability to bind a human-like sialic acid receptor. H9N2 HAs also display variability in their pH of fusion, ranging between pH 5.4 and 5.85 which is similar to that of the first wave of human H1N1pdm09 viruses but lower than the pH of fusion seen in zoonotic H5N1 and H7N9 viruses. Our results suggest possible molecular mechanisms that may underlie the relatively high prevalence of human zoonotic infection by particular H9N2 virus lineages.

## INTRODUCTION

Over the past 20 years, H9N2 avian influenza viruses (AIVs) have become enzootic in poultry throughout Asia, the Middle East and North Africa, where they have caused major economic losses to the poultry industry, as well as sporadic zoonotic human infections.^1, 2, 3, 4^ Studies to assess the mammalian transmissibility of H9N2 viruses conducted in ferrets have clearly demonstrated both direct contact and airborne transmission to naive ferrets, both when the virus was unadapted or ‘mammalian-adapted' by serial ferret passage.^5, 6, 7, 8^ Given the transmission of H9N2 viruses in the ferret model and their widespread distribution, H9N2 AIVs are considered to be potentially pandemic viruses.

Many properties of influenza virus proteins have been determined to have a role in the adaptation of avian viruses to humans. Two of the best-studied properties are haemagglutinin (HA) receptor-binding preference and the pH of fusion.^9, 10, 11^ The human upper respiratory tract is dense in α2,6-linked sialic acid (SA) while the avian respiratory and gastrointestinal tracts are abundant in α2,3-linked SA.^12^ During the adaptation to infecting and transmitting between humans, AIVs change receptor preference from ‘avian-like' α2,3-linked SA to ‘human-like' α2,6-linked SA. The molecular basis of this change in receptor preference has been previously evaluated for several influenza subtypes (e.g., H2, H3, H5, H7 and H9) and has been partially, or entirely, attributed to a single amino-acid change at residue 226 in the HA molecule from glutamine to leucine.^13, 14, 15, 16^ In an H9N2 isolate, a Q226L mutation alone allowed the virus to replicate better in human primary epithelial airway cells and L226 has also been shown to be structurally involved in the interaction with a α2,6 receptor analogue.^17, 18^ In addition to receptor binding, several studies have shown that efficient airborne transmission of AIV between ferrets requires the HA molecule to become stabilized and thus exhibit a lower pH of fusion.^9, 10, 19, 20, 21^ This increased pH stability of the HA is thought to be important for maintaining viral infectivity in the relatively harsh microenvironment of respiratory droplets and the mildly acidic environment of the mammalian nasal tract and human influenza viruses generally have a lower pH of fusion (<pH 5.5), whereas AIVs have a higher pH of fusion (>pH 5.5).^19, 22^

Based on the phylogenetic analysis of the HA gene, Eurasian H9N2 viruses are classified into three distinct lineages: (i) the BJ94 lineage, prevalent in China and Vietnam; (ii) the Y439 lineage of viruses, found in chickens in Korea; and (iii) the G1 lineage, the lineage with most widespread distribution, prevalent throughout North Africa, the Middle East and Southern China.^23^ The G1 lineage can be further divided into two discrete ‘Eastern' and ‘Western' sub-lineages: the minor ‘Eastern' G1 sub-lineage co-circulates with BJ94 lineage H9N2 viruses in South China and Vietnam, while the major ‘Western' G1 sub-lineage exists in an almost contiguous region from Bangladesh to Morocco.^1, 24^

Despite the extensive global distribution and diversity of H9N2 viruses and the potential threat to human health associated with their circulation, contemporary H9N2 viruses have not yet been extensively characterized in terms of HA receptor-binding preference and pH of fusion. Our objective of this study was to investigate these biophysical characteristics of the H9 HA protein across a range of H9N2 viruses, focussing particularly on the genetically and antigenically diverse G1 lineage.^1, 25^

## MATERIALS AND METHODS

### Cells, eggs and viruses

MDCK, 293T and Vero cells were grown in Dulbecco's modified Eagle medium with 10% foetal bovine serum. Recombinant viruses were generated using a standard eight plasmid reverse genetics (RG) system, as described elsewhere.^25, 26^ All viruses were rescued using the strain-specific HA plasmids (for highly pathogenic AIV strains, the polybasic HA cleavage sites were replaced with monobasic cleavage sites). For H9N2 and H5N1 viruses, respectively, the NA proteins were replaced with either the N2 NA of A/chicken/Pakistan/UDL-01/2008 (for H9N2 viruses) or N1 NA plasmid of A/turkey/Turkey/1/2005 (for H5N1 viruses). The six remaining internal gene segments were from A/Puerto Rico/8/34 (PR8; Table 1). RG viruses were propagated in 10-day-old embryonated eggs or MDCK cells.

### Virus purification

Viruses were purified and quantified using the well-established methods.^27^ Virus from allantoic fluid or cell culture supernatants was pelleted by ultracentrifugation for 2 h at 135 000 × *g* at 4 °C. Virus was then resuspended and purified through a 30%–60% continuous sucrose gradient, again for 2 h at 135 000 × *g* at 4 °C. Virus was subsequently diluted in phosphate-buffered saline and pelleted by ultracentrifugation and resuspended in phosphate-buffered saline. Virus concentrations were estimated by comparative densitometry of nucleoprotein by sodium dodecyl sulphate-polyacrylamide gel electrophoresis and by nucleoprotein enzyme-linked immunosorbent assay.^27^

### Bio-layer interferometry

Binding of purified virus to sialylated receptor analogues was measured using an Octet RED bio-layer interferometer (Pall FortéBio, California, CA, USA) as previously described.^27^ Receptor analogues used were sialoglycopolymers consisting of a 30 kDa polyacrylamide backbone conjugated to 20 mol% trisaccharides, α2,6-sialyllactosamine (6SLN), α2,3-sialyllactosamine (3SLN) or Neu5Ac α2,3Gal β1-4(6-HSO_3_)GlcNAc (3SLN(6su)) and 5 mol% biotin (Lectinity). Sialoglycopolymers were immobilized onto streptavidin-coated biosensors (Pall FortéBio) at concentrations ranging from 0.01 to 0.5 μg/mL in 10 mM HEPES, pH 7.4, 150 mM NaCl, 3 mM EDTA and 0.005% Tween-20 (HBS-EP). Virus was diluted to a concentration of 100 pM in HBS-EP containing 10 μM of the neuraminidase inhibitors oseltamavir carboxylate (Roche, Welwyn Garden City, UK) and zanamivir (Sigma-Aldrich, Gillingham, UK). Virus association with the bound receptor analogues was measured at 20 °C for 30 min. Virus-binding amplitudes were normalized to fractional saturation of the sensor surface and plotted against sugar loading. These fractional saturation curves were well fitted by a variation of the Hill equation as described in previous studies.^28, 29^ Dissociation constants (*K*_D_) values for virus binding at any sugar loading could then be calculated using the following standard equation, *K*_D_=([Virus]−*f* × [Virus])/*f*, where [Virus] is the virus concentration and *f* is the fractional saturation.^28, 29^ Relative *K*_D_ values were then calculated.

### Syncytium-formation assay

Monolayers of Vero cells were infected with virus at a multiplicity of infection of three virus TCID_50_ (median tissue culture infective dose) per cell. At 16 h postinfection, cells were treated with 5 μg/mL of TPCK trypsin diluted in serum-free medium and overlayed for 10 min with 2-(*N*-morpholino)ethanesulfonic acid buffers adjusted across a range of pHs (pH 4.8–6.2). Cells were incubated for 3 h at 37 °C to allow for syncytium formation. Cells were fixed with methanol:acetone (1:1 v/v) and then treated with Giemsa stain, modified solution (Sigma-Aldrich). Images were taken on the EVOS XL cell imaging system (Life Technologies, Thermo Fisher Scientific, Paisley, UK). To quantify syncytium formation, five random fields were photographed and the proportion of nuclei in syncytia over total nuclei in each field was determined. Asymmetric sigmoidal five-parameter dose–response curves were then modelled onto the values using Graphpad Prism 6 (GraphPad Software, La Jolla, CA, USA) and the point where 50% of maximum syncytium formation was estimated was taken as the predicted pH of fusion.

## RESULTS

### Receptor-binding characteristics of H9N2 viruses

To investigate the receptor binding of different H9N2 viruses, we selected 12 representative HAs from field isolates containing variation within, or nearby the receptor-binding site (RBS), for example, at positions 190, 226 and 217 (H3 numbering) all known to influence H9N2 receptor binding^17, 30, 31^ (Table 1). We then generated RG viruses containing the internal genes of A/Puerto Rico/1/34 (PR8) with the respective wild-type H9 HA genes, together with their subtype-specific neuraminidases. We utilized bio-layer interferometry to characterize the receptor-binding profiles of these RG viruses alongside several human and AIVs, in a manner similar to that previously described.^27, 28, 29, 32, 33, 34^ Using bio-layer interferometry, the relative estimated dissociation constant (*K*_D_) values for virus binding could be estimated in order to quantitatively compare the binding avidity of different viruses to the receptor analogues. We measured binding to the avian and human receptor analogues, 3′-sialylacetyllactosamine (3SLN) and 6′-sialylacetyllactosamine (6SLN), as well as to a sulphated version of the avian analogue 3SLN, Neu5Ac α2,3Gal β1-4(6-HSO_3_)GlcNAc (hereafter referred to as 3SLN(6su)) that has additionally been implicated in AIV receptor binding.^35, 36^

**Figure 1:**
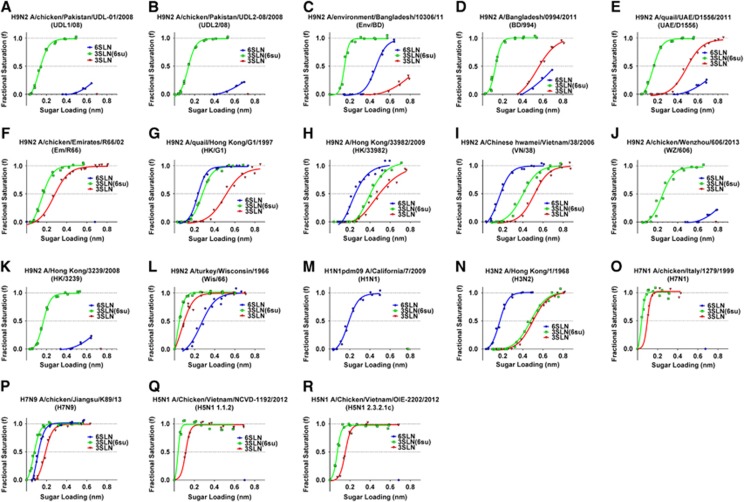
Receptor-binding properties of H9N2 haemagglutinins. Receptor-binding properties of different influenza viruses (indicated as **A**–**R**) were tested by bio-layer interferometry. Virus binding was measured for three different receptor analogues: α2,3-SLN (3SLN, shown in red), sulphated α2,3-SLN (3SLN(6su), shown in green) and α2,6-SLN (6SLN, shown in blue). Data are the combination of two repeats for each virus and receptor analogue combination.

All H9N2 RG viruses tested showed stronger binding to the 3SLN(6su) analogue than to the non-sulphated form (Figures 1A–1L and Table 1). The ‘Western' G1 sub-lineage viruses, A/chicken/Pakistan/UDL-01/2008 (UDL1/08), A/chicken/Pakistan/UDL-02/2008 (UDL2/08), A/environment/Bangladesh/10306/2011 (Env/BD), A/Bangladesh/0994/2011 (BD/994) and A/quail/United Arab Emirates/D1556/2011 (UAE/D1556), as well as the two BJ94 lineage viruses examined, A/chicken/Wenzhou/606/2013 (WZ/606) and A/Hong Kong/3239/2008 (HK/3239), showed >150-fold binding preference towards 3SLN(6su) than to either 6SLN or 3SLN, as determined by relative estimates of *K*_D_ for virus binding (Figures 1A–1E, 1J and 1K and Table 1). A/chicken/Emirates/R66/2002 (Em/R66) whose HA gene lies phylogenetically between the ‘Eastern' and ‘Western' G1 HA sub-lineages, showed no detectable 6SLN binding and bound to the sulphated avian analogue >5-fold stronger than to 3SLN (Figure 1F and Table 1). Both H5N1 viruses, as well as the H7N9, H7N1 and H3N2 viruses, also showed a preference for the sulphated over the non-sulphated avian receptor, with differences in the estimated relative *K*_D_ between 2- and 160-fold (Figures 1N–1R and Table 1). Conversely, the three ‘Eastern' G1 sub-lineage RG viruses, A/quail/Hong Kong/G1/1997 (HK/G1), A/Hong Kong/33982/2009 (HK/33982) and A/Chinese hwamei/Vietnam/38/2006 (VN/38), showed up to a 55-fold difference between binding avidity towards 6SLN, the human-like receptor analogue, over the next highest binder, 3SLN(6su). These viruses displayed a receptor-binding preference similar to that seen in the 1968 pandemic H3N2 virus, though not to the same extent of pandemic H1N1 2009 swine origin influenza, which bound solely 6SLN (Figures 1G–1N and Table 1). Among the human H9N2 RG viruses tested (HK/33982, HK/3239 and BD/994), only HK/33982 of the ‘Eastern' G1 sub-lineage showed any measurable binding avidity towards 6SLN.

6SLN binding has previously been attributed to the presence of leucine at position 226 (H3 numbering, 216 in mature H9 HA numbering, that is, counting from after the HA signal peptide) of the RBS of HA in multiple subtypes.^13, 14, 15, 17^ However, we found (Table 1) that there was no apparent correlation between 6SLN-binding avidity and the presence of leucine or glutamine at position 226: only one of the six tested H9N2 RG viruses with L226 had any appreciable 6SLN binding compared with three of the six of Q226-containing viruses.

We further found that the amino acid at position 190 (180 in mature H9 HA numbering), known to be important in H1 HA receptor-binding preference,^13^ appeared to show some correlation with 6SLN-binding avidity. Viruses that have an acidic residue (D/E) at 190 bound to 6SLN better than viruses with aliphatic residues (A/I) with 3/4 and 0/7 6SLN binders, respectively.

Position 227 (217 in mature H9 HA numbering) has previously been shown to be important in H9N2 receptor binding;^31^ when position 190 is considered alongside the presence or absence of glutamine at position 227, we found that all H9N2 viruses included in this study clustered into two groups: all four viruses with an acidic residue at 190 paired with a glutamine at position 227 showed binding to 6SLN, whereas the remaining 8 H9N2 viruses displayed little or no detectable binding to this analogue.

As observed by others previously,^35^ we found that an absence of a negatively charged residue at position 190 showed a correlation with a strong preference towards sulphated 3SLN over the non-sulphated form. All 5 viruses with glutamic or aspartic acid at 190 showed only a moderate binding preference for 3SLN(6su) over 3SLN of between 3- and 37-fold. Conversely, of the remaining 7 viruses with aliphatic residues at 190, all had >180-fold binding preference towards the sulphated receptor analogue when compared with the non-sulphated (Table 1). This result reiterates the potential importance of position 190 for H9 receptor-binding preference.

### pH of fusion of H9N2 HAs

As well as receptor-binding preference, the adaption from transmission between avian hosts to transmission between humans is thought to involve changes in the pH of fusion of the HA molecule, with a switch from an HA that fuses at a higher pH to an HA that fuses at a lower pH.^9, 10, 22^ We characterized the pH of fusions for each of the HAs that have been tested for receptor binding by bio-layer interferometry and six additional H9 HAs that possessed identical RBS sequences to those previously tested. We used a syncytium-formation assay to estimate of the pH of fusion for each HA.^19^

**Figure 2:**
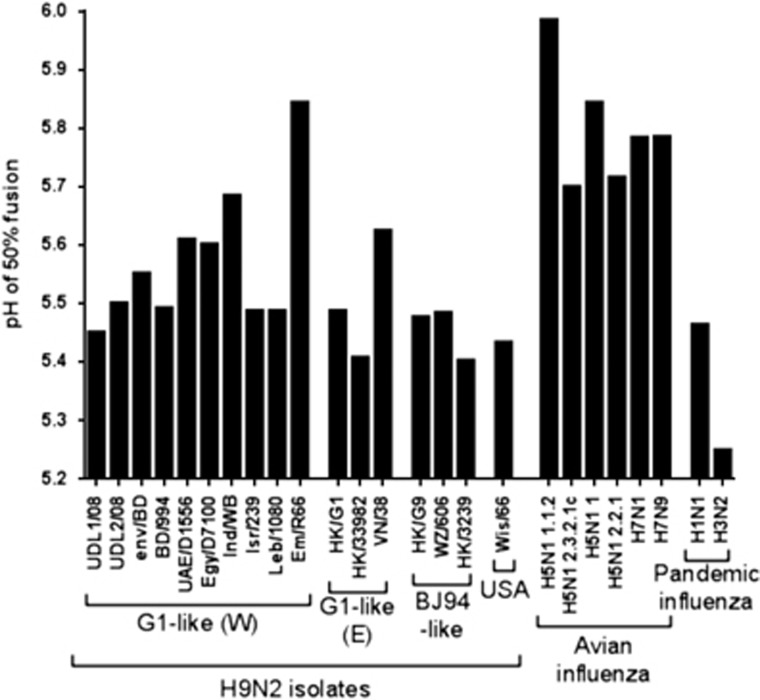
pH of fusion of influenza viruses. pH of fusion of different H9 and non-H9 influenza viruses was estimated using syncytium formation by virus-infected Vero cells across a range of pH values following trypsin activation of haemagglutinin. Values indicate pH where 50% of maximum syncytium formation was observed.

We initially modelled sigmoidal dose–response curves onto our syncytium-formation data showing consistently good fits of data (mean *R*^2^ value=0.968, range 0.924–0.994; Supplementary Figure S1). Using selected human and AIV controls, we established that our assay yielded results consistent with previously reported pH of fusion (Figure 2).^19, 37, 38, 39^ All avian H5 and H7 viruses had relatively high pH of fusion (between pH 5.7 and 6.0), while the pandemic isolates A/California/7/2009(H1N1) and A/Aichi/2/68(H3N2) exhibited lower pH of fusion between pH 5.2 and 5.5, typical of human adapted influenza viruses.^19^ The H9N2 viruses examined in this study had pH of fusion that ranged between 5.41 and 5.84. Although most H9N2 viruses had pH of fusion between 5.4 and 5.6, the ‘Western' G1 sub-lineage HAs Em/R66 and A/chicken/India/WB-NIV1057169/2010 (Ind/WB) had higher pH of fusion values of 5.84 and 5.68, respectively. The three human H9N2 isolates tested (BD/994, HK/3239 and HK/33982) had among the lowest pH of fusion, between 5.4 and 5.5 (Figure 2). Overall, currently circulating H9N2 HAs tended to have a pH of fusion intermediate between H5 and H7 viruses and human adapted pandemic influenza viruses.

## DISCUSSION

In this study, we assessed the receptor-binding characteristics and pH of fusion, two correlates of zoonotic and pandemic potential of AIVs, of a number of contemporary and some historical H9N2 isolates.^9, 10, 19^ This work is the first to take a biophysical approach characterizing H9N2 virus receptor-binding preference, while several previous studies have investigated receptor-binding preference using glycan microarrays,^40^ enzyme-linked immunosorbent assay-based methods^5, 30, 35, 41, 42, 43, 44^ or through direct mammalian infectivity/transmission *in vivo*.^5, 8, 45^ To our knowledge, this is the first report that assesses pH of membrane fusion of H9N2 HAs.

In contrast to H5 and H7 subtypes, which bind well to the avian receptor analogue 3SLN,^28, 29, 35, 46^ we found that the majority of contemporary H9N2 HAs had no detectable binding to 3SLN. However, all H9N2 viruses tested bound 3SLN(6su), a sulphated derivative of 3SLN. Sulphated, sialylated glycans have been suggested as potential host receptors for avian influenza binding, including previously circulating H9N2 influenza strains;^35, 36, 46, 47^ our work further supports a role for these glycans as receptors in poultry for contemporary H9N2 AIVs. However, the abundance and distribution of sulphated, sialylated glycans in either mammalian or avian species remains poorly characterized. Several recently published studies have used the receptor analogues 3SLN and 6SLN to estimate the zoonotic potential of contemporary H9N2 viruses and, in many cases, have concluded that these viruses bind preferentially to 6SLN.^5, 30, 31, 42^ As found for older H9N2 isolates by Gambaryan *et al*,^35^ we found currently that circulating H9N2 isolates from the G1 and BJ94 lineages continue to display a strong preference for binding 3SLN(6su), in the absence of any non-sulphated 3SLN binding; furthermore binding to 3SLN(6su) was appreciably stronger than to 6SLN. It is therefore important for this sulphated version of the avian receptor analogue to be included in future studies on the receptor preference of H9N2 and other AIVs.

We demonstrate that the ‘Eastern' G1 sub-lineage, represented by the viruses HK/33982 (2009), VN/38 (2006) and HK/G1 (1997), displayed a preference in binding to the human-like receptor 6SLN, similar to that seen in pandemic H3N2 virus and previously described for the older isolate HK/G1;^35, 43^ however, here we show that two more recent viruses of this ‘Eastern' lineage continue to possess this human receptor preference to an even greater extent. This suggests an explanation for the high frequency of detection of viruses of this sub-lineage in humans. This 6SLN preference is intriguing as these viruses might have a fitness disadvantage in species where α2,6-linked sialic acids are rare, whereas α2,3-linked sialic acids are abundant, for example, chickens.^48^ Therefore, we speculate that minor poultry species such as quail, which have been found to contain abundant α2,6-linked receptors^49, 50, 51^ and harbour viruses that have a preference towards 6SLN,^52, 53^ may be the natural host for these viruses. This hypothesis is further supported by the abundance of viruses of this sub-lineage isolated from quails and quail markets in China and Hong Kong (60% of these viruses were isolated from quails, *n*=58).^54, 55^

Amino acids at positions 190, 226 and 227 (H3 numbering) have each been implicated in H9 HA receptor-binding preference.^17, 31, 35^ For G1 lineage viruses, the current most common combination is 190A, 226L and 227I (ALI), as seen in UDL1/08, UDL2/08 and BD/0994. This combination is shared by >74% (*n*=345 virus sequences obtained from GenBank) of post-2008 H9N2 viruses of this lineage. The results described here do not support an association of this amino-acid combination with significant 6SLN binding. In contrast, Chinese BJ94 lineage viruses exhibited a much larger range of amino-acid combinations at these residues in the RBS, with the most common motifs being TQQ, ALQ (present in HK/3239), VLQ and ALM (present in WZ/606) with post-2008 incidences of 31.2%, 19.5%, 13.9% and 13.4%, respectively (*n*=1087). No BJ94 lineage HAs have been isolated since 2008 that contain E190 in the RBS. Given these observed binding preference, we suggest that these lineages may pose a relatively low zoonotic threat when receptor binding alone is considered.

In contrast to what was expected from structural and mutagenesis experiments,^17, 18^ we found that position 226 of the H9 HA, a commonly used marker of zoonotic potential, does not correlate with an avidity for human-like receptors (Table 1). Instead, we found a strong positive correlation between the presence of acidic residues at position 190 and 6SLN binding. Furthermore, when position 190 is considered alongside position 227, all 12 H9N2 viruses fell neatly into two groups: viruses with 190E/D and 227Q (*n*=4) displayed appreciable 6SLN binding, whereas the remaining 8 viruses did not. These results suggest the need to look beyond residue 226 when assessing the impact of amino acids in the RBS. Our results support the hypothesis previously posited by Gambaryan *et al.*^35^ that aliphatic, rather than acidic residues at position 190, altered the binding preference of H9 HAs towards sulphated 3SLN.

We have shown that H9N2 HAs generally have a lower pH of fusion compared with H5 and H7 AIVs and that their range of pH of fusion overlaps with those of early human H1N1pdm09 isolates and ferret transmissible H5N1 viruses.^10, 19, 56^ Interestingly, H9N2 HAs isolates from humans had among the lowest pH of fusion of all the viruses tested. The low pH of fusion of these HAs correlated better as a marker of the zoonotic viruses than 6SLN binding as two of the three human H9N2 isolates investigated in this study showed negligible binding to the human-like receptor analogue. It is impossible, however, to distinguish whether these human H9N2 acquired an intrinsically more stable HA during replication and adaption in the human upper respiratory tract or whether these properties were already present in the parental viruses that initiated the infections. Viruses from the ‘Eastern' G1 sub-lineage showed relatively low pHs of fusion compared with other AIV subtypes (between 5.4 and 5.6) and 6SLN binding, suggesting that they have enhanced potential infectivity and transmissibility in humans compared with the other H9N2 lineages. The molecular determinants of the pH of fusion of influenza HAs is multifaceted and can be influenced by substitutions in the fusion peptide, the HA trimer interface, the HA RBS and changes in HA glycosylation.^57, 58, 59^ Further characterization of closely related HAs with widely different pH stabilities and additional *in vivo* studies are required to better understand the biological significance of this observed variation in the pH of fusion.

Although the experiments described in this study were limited to *in vitro* characterizations, several of the H9N2 recombinant viruses used in this work were previously evaluated for their mammalian infectivity and transmissibility in the ferret model.^8, 45^ In separate studies by Wan *et al*^8^ and the Saint Jude's Centre for Excellence for Influenza Research and Surveillance (SJCEIRS) H9 working group,^45^ the ‘Eastern' G1 sub-lineage human isolate HK/33982, as well as a virus similar in sequence to HK/G1, were shown to infect and transmit efficiently to contact ferrets, although not by respiratory droplets. HK/33982 was additionally shown to replicate to a high titre in human primary bronchial epithelial cells.^43^ Both these properties of replicative fitness and transmissibility in ferrets may be explained in part by the strong receptor-binding preference towards 6SLN and a low pH of fusion. Furthermore, several BJ94 lineage viruses that are closely related to the viruses used in this study were previously described by Li *et al*^5^ as having the ability to transmit by airborne droplets in ferrets. In our study, both BJ94 lineage viruses had poor 6SLN binding but low pH of fusion, thus suggesting that HA stability alone may be sufficient to support airborne transmission in the ferret model.

In conclusion, we have analysed the receptor-binding characteristics and pH of fusion of a range of H9 HAs and discussed the potential relevance of these biomarkers for zoonotic risk assessments. Our results indicate that, based on the two properties tested, the H9 HAs with the highest zoonotic potential may be those of the G1 ‘Eastern' sub-lineage that currently circulate in Southern China and Vietnam. The BJ94 lineage and ‘Western' G1 sub-lineage viruses evaluated in this study show low human-like receptor-binding preference but have pH of fusion similar or lower than that of early human H1N1pdm09 isolates,^19^ suggesting that these viruses could also adapt to humans with relatively few additional mutations. Overall, our study contributes to the body of literature that combines the use of molecular, biophysical and virological indicates for system risk assessments of AIV zoonotic and pandemic potential.

## Figures and Tables

**Table 1:** Receptor binding and pH of fusion of viruses investigated in this study

**Virus name**	**Alias**	**Lineage/clade**	**Host species**	**Receptor-binding site**^a^			**Estimated pH of fusion**	**Relative** ***K***_**D**_ **values to receptor analogues**^b^		
				**190**	**226**	**227**		**6SLN**	**3SLN**	**3SLN(6su)**
*H9N2 isolates*										
A/chicken/Pakistan/UDL-01/2008	UDL1/08	G1 (W^c^)	Chicken	A	L	I	5.46	>^d^	>	2.2
A/chicken/Pakistan/UDL-02/2008	UDL2/08	G1 (W)	Chicken	A	L	I	5.50	>	>	2.6
A/environment/Bangladesh/10306/2011	Env/BD	G1 (W)	Quail	A	Q	T	5.54	690	>	4.0
A/Bangladesh/0994/2011	BD/994	G1 (W)	Human	A	L	I	5.49	>	1600	2.1
A/quail/United Arab Emirates/D1556/2011	UAE/D1556	G1 (W)	Quail	I	Q	F	5.64	>	890	4.8
A/chicken/Egypt/D7100/2013	Egy/D7100	G1 (W)	Chicken	A	L	I	5.61		ND^e^	
A/chicken/India/WB-NIV1057169/2010	Ind/WB	G1 (W)	Chicken	A	L	I	5.68		ND	
A/chicken/Israel/239/2013	Isr/239	G1 (W)	Chicken	A	L	I	5.49		ND	
A/chicken/Lebanon/1080/2004	Leb/1080	G1 (W)	Chicken	A	L	Q	5.49		ND	
A/chicken/Emirates/R66/2002	Em/R66	G1 (W)	Chicken	E	Q	L	5.84	>	24	4.4
A/quail/Hong Kong/G1/1997	HK/G1	G1 (E)	Quail	E	L	Q	5.48	31	1000	67
A/Hong Kong/33982/2009	HK/33982	G1 (E)	Human	D	Q	Q	5.43	35	930	400
A/Chinese hwamei/Vietnam/38/06	VN/38	G1 (E)	Passerine	D	Q	Q	5.62	6.4	1100	350
A/chicken/Hong Kong/G9/1997	HK/G9	BJ94	Chicken	A	L	Q	5.48		ND	
A/chicken/Wenzhou/606/2013	WZ/606	BJ94	Chicken	A	L	M	5.48	>	>	38
A/Hong Kong/3239/2008	HK/3239	BJ94	Human	A	L	Q	5.41	>	>	6.9
A/turkey/Wisconsin/1/1966	Wis/66	USA	Turkey	E	Q	Q	5.44	9.8	0.14	0.004
*Non-H9N2 viruses*										
A/California/7/2009	H1N1	pdm09	Human	D	Q	E	5.46	11	>	>
A/Aichi/2/1968	H3N2	NA	Human	E	L	S	5.25	1	97	42
A/chicken/Italy/1279/1999	H7N1	Eurasian	Chicken	E	Q	S	5.78	>	0.65	0.01
A/Shanghai/02/2013	H7N9	Eurasian	Human	E	L	S	5.76	0.8	11	0.16
A/chicken/Vietnam/NCVD-1192/2012	H5N1 1.1.2	1.1.2	Chicken	E	Q	S	5.99	>	1.2	0.008
A/chicken/Vietnam/OIE-2202/2012	H5N1 2.3.2.1c	2.3.2.1c	Chicken	E	Q	S	5.70	>	4.8	0.17
A/chicken/Vietnam/1194/2004	H5N1 1	1	Chicken	E	Q	S	5.85		ND	
A/turkey/Turkey/1/2005	H5N1 2.2.1	2.2.1	Turkey	E	Q	S	5.72		ND	

Abbreviations: not available, NA; not determined, ND.

a Selected receptor-binding site residues shown based on location and variability, H3 numbering used throughout.

b Relative *K*_D_ calculated with H3N2 virus binding to 6SLN set equal to 1, values >1 indicate weaker binding, while values <1 indicate stronger binding.

c (W) or (E) indicates whether viruses belong to Western or Eastern G1 sub-lineage.

d > indicates values >2 000, which cannot be accurately quantified.

e ND indicates viruses for which only the pH of fusion was measured.
