# Supplementary material for: Variability in H9N2 haemagglutinin receptor-binding preference and the pH of fusion
Source: Emerg Microbes Infect. 2017 Mar 22;6(3):e11–. doi: 10.1038/emi.2016.139 (PMC5378918; doi:10.1038/emi.2016.139)
Supplement: Supplementary Information [file emi2016139x1.pdf]

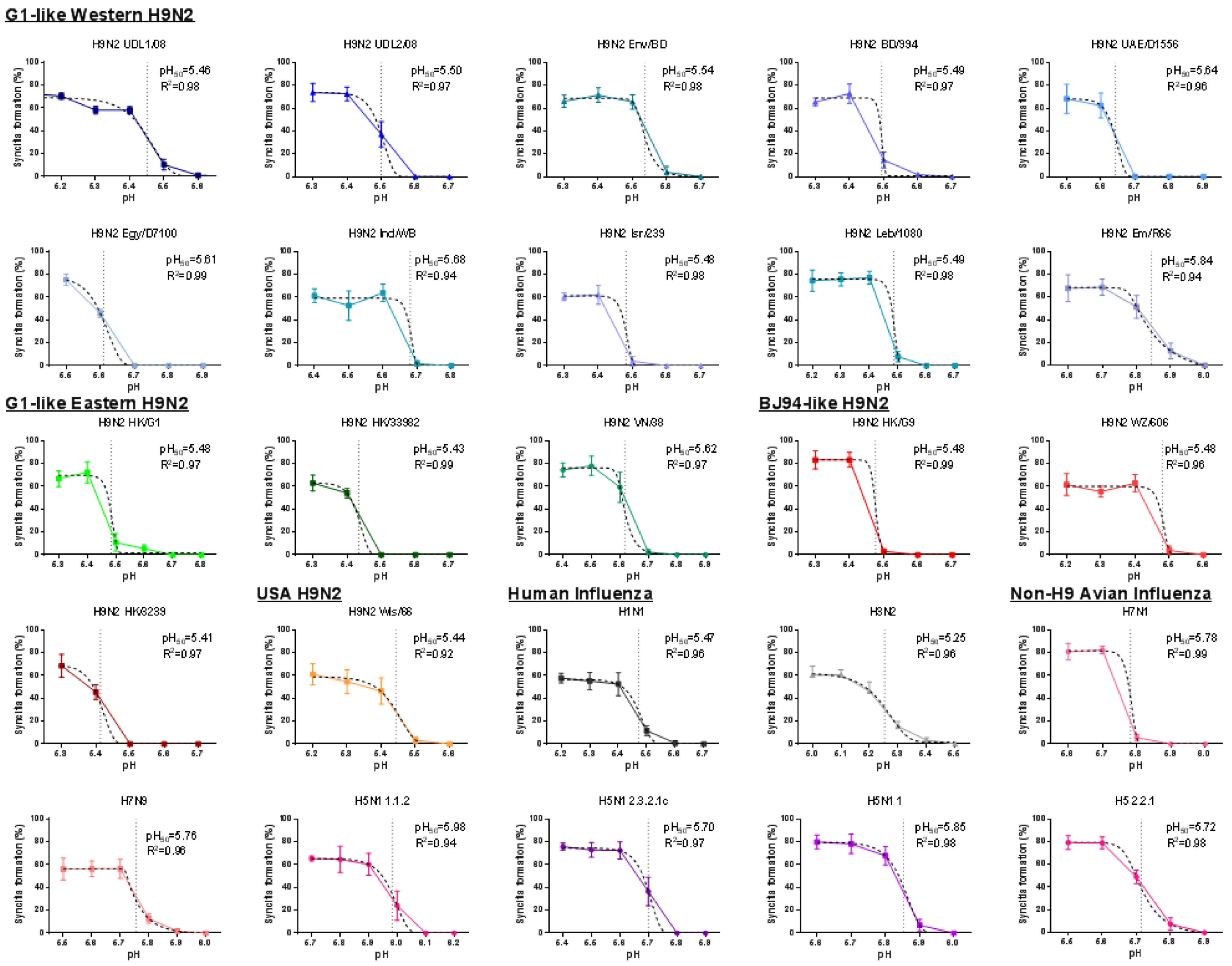

Supplementary Figure S1 Individual plots of syncytium formation used to predict the pH of 50% fusions. Cells were infected with a high MOI (3 TCID50/cell) of virus. 16 hours post-infection cells were treated with TPCK trypsin to active the HA and then treated with a range of pH buffers. Three hours post-fusion cells were fixed and Giemsa stained. Images of five random fields were used to quantify syncytium formation for each virus. Sigmoidal dose-response curves were then modelled to the data and used to estimate pHs of 50% fusion. R2 of the sigmoidal curves were also calculated and are shown for each plot.
